# The Multifunctional Roles of Polyphenols in Plant-Herbivore Interactions

**DOI:** 10.3390/ijms22031442

**Published:** 2021-02-01

**Authors:** Sukhman Singh, Ishveen Kaur, Rupesh Kariyat

**Affiliations:** 1 Department of Biology, University of Texas Rio Grande Valley, Edinburg, TX 78539, USA; sukhman.singh01@utrgv.edu; 2 School of Earth, Environmental and Marine Sciences, University of Texas Rio Grande Valley, Edinburg, TX 78539, USA; Ishveen.kaur01@utrgv.edu

**Keywords:** secondary metabolites, polyphenols, phenylpropanoid pathway, phenolic acid, flavonoids, lignans, insect herbivores, chemical defenses

## Abstract

There is no argument to the fact that insect herbivores cause significant losses to plant productivity in both natural and agricultural ecosystems. To counter this continuous onslaught, plants have evolved a suite of direct and indirect, constitutive and induced, chemical and physical defenses, and secondary metabolites are a key group that facilitates these defenses. Polyphenols—widely distributed in flowering plants—are the major group of such biologically active secondary metabolites. Recent advances in analytical chemistry and metabolomics have provided an opportunity to dig deep into extraction and quantification of plant-based natural products with insecticidal/insect deterrent activity, a potential sustainable pest management strategy. However, we currently lack an updated review of their multifunctional roles in insect-plant interactions, especially focusing on their insect deterrent or antifeedant properties. This review focuses on the role of polyphenols in plant-insect interactions and plant defenses including their structure, induction, regulation, and their anti-feeding and toxicity effects. Details on mechanisms underlying these interactions and localization of these compounds are discussed in the context of insect-plant interactions, current findings, and potential avenues for future research in this area.

## 1. Introduction

The millions of years of dynamic co-existence and relentless competition for survival has led plants to evolve complex strategies to survive against the onslaught of damaging insect herbivores [1,2,3], primarily mediated through tolerance and resistance mechanisms [4,5]. While resistance traits assist plants to prevent the attack of insects, tolerance allows them to combat herbivory or offset fitness consequences by increasing the photosynthetic activity at the damaged site, and utilization of stored resources for compensatory growth [4]. Resistance mechanisms also include mechanical defenses to deter the insects from feeding- using morphological adaptations including, but not limited to waxy cuticle, trichomes, thorns, and spines [6,7]. Being the first line of defense, herbivores have to face these challenges pronto as they come in contact with plants, although these defenses can also act in tandem to successfully ward off herbivory [4]. However, selection pressure for survival in this never ending co-evolutionary arms race has also led to the development of complex, biochemically based, and tightly regulated second line of defenses. These include the production of toxins that deter herbivores from feeding, reduce the palatability/digestibility of plant tissue, and compounds that can negatively affect herbivore growth and development [8,9]. These defenses also include the release of constitutive and herbivore induced plant volatiles that attract predators and parasitoids, and in many cases, selectively repel herbivores [10,11]. Collectively, these compounds are defined as plant secondary metabolites.

Plant cells produce two types of compounds; primary metabolites and secondary metabolites (plant secondary metabolites; PSM) [12,13]. Primary metabolites include compounds vital for plant growth, development, and fitness. These include carbohydrates, lipids, nucleic acids, and proteins inevitable for cell structure, and physiological and biochemical functioning in plants; whereas PSM, although not directly involved in growth and metabolism, are essential for interactions with the environment. They are synthesized and can also be induced during biotic and abiotic stresses- protecting the plant from insects, mammalian herbivores, micro-organisms, UV radiation, high temperature, shading, mechanical injury, wounding, and heavy metal toxicity to name a few [14,15,16,17]. As an anti-herbivore defense, they also improve host plant survival and fitness [12,18,19] by negatively affecting the survivability, vigor, host location and fitness of the herbivores [8,20,21,22,23]. Furthermore, the production of these compounds is tightly regulated and decreases once they regain normal state post induction, since they require huge investment of resources; making it expensive to continuously produce them, leading to growth-fitness trade-offs [4,24,25].

## 2. Classification

**Figure 1:**
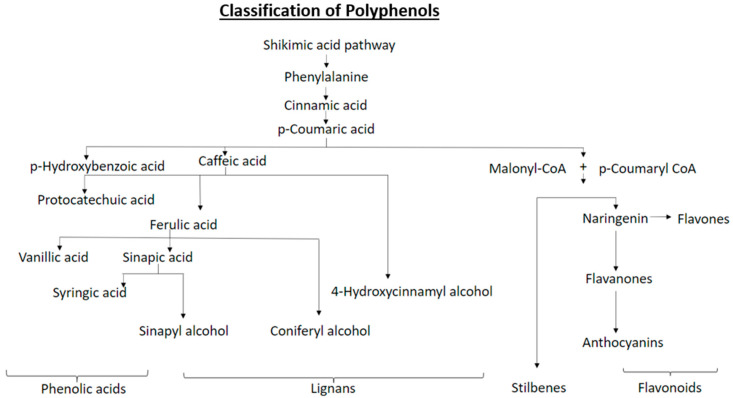
Basic classification and synthesis outline of major polyphenol classes in plants.

Plant secondary metabolites are generally divided into three broad classes: terpenoids, phenolics and alkaloids, with phenolics (polyphenols) being the largest, diverse and most widely distributed class among them. Several thousand polyphenolic compounds are found in plants, synthesized via the shikimic acid-derived phenylpropanoid and/or polyketide pathways [26]. They have a basic structure consisting of benzene ring with a hydroxyl group attached, without any nitrogen-based functional group [27,28,29]. L-phenylalanine is the primary compound in this pathway to be synthesized and form the basis for downstream synthesis of other polyphenols (Figure 1). Major groups of polyphenols include flavonoids (C_6_-C_3_-C_6_), phenolic acids (C_6_-C_1_), stilbenes (C_6_-C_2_-C_6_) and lignans (C_6_-C_3_). Polyphenols not only contribute to the flavor, color, odor, astringency, oxidative stability and bitterness [30,31] of different plant parts, but also play a critical role as plant chemical defenses [30].

The idea of plant-insect interactions either positively or negatively affected by polyphenols was first proposed by Fraenkel in 1959 [32]. Following that, numerous studies investigated the defensive as well as stimulatory roles of such metabolites on insect herbivores. For instance, grain aphid (*Sitobion avenae* F.) infestation in winter triticale (*Triticosecale* Wittm) seedlings induces bioactive compounds such as phenolic acids that provide resistance against them [33]. Kariyat et al. (2019) showed that 3-deoxyanthocynadin (flavonoids) present in wild type sorghum (*Sorghum bicolor* (L.) Moench Family: Graminaceae) caused significantly higher mortality and reduced population growth in corn leaf aphid (*Rhopalosiphum maidis* Fitch), when compared to null mutants devoid of them [23]. More recently, we also showed that polyphenol-rich pericarp extract of purple corn (*Zea mays*) negatively affected growth, development and adult fitness traits in tobacco hornworm (*Manduca sexta* L.), a specialist herbivore on Solanaceae [8,22]. Consistent with these observations, it has been well documented that different groups of polyphenols collectively protect most plant species against wide range of attackers. For example, chlorogenic acids in chrysanthemum (*Dendranthema grandiflora* (Ramat.) effectively defend against thrips [34], pisatin (flavonoid) deters pea-aphid (*Acyrthosiphon pisum* Harris) in pea [35] (Table 1), and ferulic acid in rice impart resistance against brown planthopper (*Nilaparvata lugens* Stål) [36] to name a few. Increase in phenolic acids and flavonoids, especially quercetin has also been observed in white cabbage (*Brassica oleraceae* L. *var. capitata f. alba*; Family: Brassicaceae) upon infestation by cabbage butterflies (*Pieris brassicae* L.) and flea beetles (*Phyllotreta nemorum* L.) [37]. A detailed version of similar examples and effects of polyphenols are described in Table 1. Taken together, it is clear that polyphenols (the primary group of PSM’s) can not only protect plants against broad spectrum of insect herbivores but can also be specific and highly regulated based on particular host-herbivore system and genotype X environment interactions- clearly warranting further in-depth look at synthesis, distribution and role in plant-herbivore interactions, the focus of this review.

## 3. Biosynthesis of Polyphenols

In plants, polyphenols are synthesized through the phenylpropanoid pathway [69]. The first compound in this pathway is L-phenylalanine, synthesized by the phenylpropanoid shikimate pathway [70]. The first step towards formation of phenolic acids is cinnamic acid synthesis by the action of phenylalanine ammonia-lyase (PAL) on L-phenylalanine [71]. Further, cinnamic acid 4-hydroxylase catalyzes cinnamic acid to form *p*-coumaric acid. Cinnamic acid also leads to the formation of *o*-Coumaric acid, which then give rise to salicylic acid → genitisic acid → *o*-Pyrocatechuic acid. *p*-Coumaric acid further leads to the formation of caffeic acid and *p*-hydroxybenzoic acid as well, which is a derivative of hydroxybenzoic acid [72]. Ferulic acid is formed by methylation of caffeic acid. Further methylation of ferulic acid give rise to sinapic acid (Figure 1). However, the formation of benzoic acid derivatives is more complex. Either they are produced from derivatives of cinnamic acid or directly from the intermediary compounds formed in the shikimate pathway [73]. Vanillic acid can be formed from ferulic acid, syringic acid from sinapic acid, protocatechuic acid from caffeic acid or *p*-hydroxybenzoic acid. Gallic acid, protocatechuic acid, vanillic acid and syringic acid can be also synthesized from hydroxybenzoic acid [74]. These phenolic acids are commonly found in the families Apiaceae, Asteraceae, Fabaceae, Moraceae, Rosaceae, Rubiaceae and Solanaceae which play significant roles as an anti-herbivore defense [29] (Table 2).

**Figure 2:**
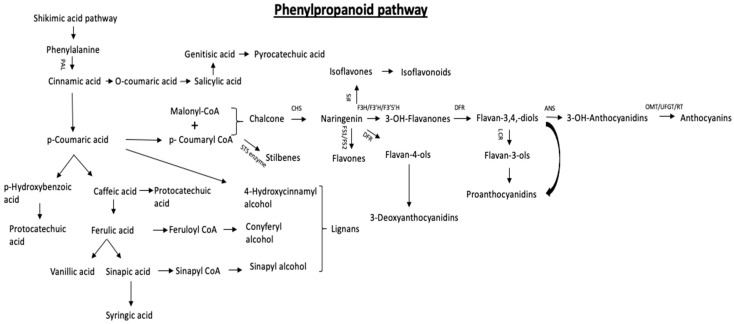
Schematic of phenylpropanoid pathway leading to synthesis of different polyphenols i.e., phenolic acids, flavonoids, stilbenes and lignans by the action of various enzymes.

Biosynthesis of flavonoids are initiated with one *p*-Coumaroyl-CoA and three malonyl-CoA molecules, where *p*-Coumaroyl-CoA is synthesized from phenolic acids in the pathway. Further, involvement of different enzymes at different levels alter the structure of the compounds formed at different levels of the pathway. The very first enzyme involved in the formation of basic flavonoid skeleton is the C-15 compound, chalcone synthase (CHS). The other various enzymes involved in pathway are chalcone isomerase (CHI), flavonoid 3-hydroxylase (F3H), flavanol synthase (FLS), dihydroflavonol reductase (DFR), anthocyanin synthase (ANS)/glucose transferase (UGTS) leading to the formation of naringenin, dihydroflavonols, flavanols, leucoanthocyanidins and anthocyanins, respectively [75,76,77] (Figure 2). Flavonoids are commonly found in angiosperms, gymnosperms and pteridophytes [75,76].

Stilbenes are also synthesized by the phenylpropanoid biosynthetic pathway. Stilbene synthase is the main enzyme required for stilbene biosynthesis and stilbene synthase gene (*STS* gene) is responsible for synthesis of these enzymes [78]. Generally, one *p*-Coumaroyl-CoA and three malonyl-CoA molecules lead to the synthesis of stilbenes by action of STS enzyme [79] (Figure 2). Stilbenes are most commonly found in Vitaceae [80], Fabaceae, Pinaceae [81,82], Gnetaceae [83], Polygonaceae [84], Ericaceae [85], and in different plant parts including leaves, roots, fruits, bark, and stem (Table 2).

Finally, lignans are also synthesized through the phenylpropanoid pathway. Coniferyl alcohol, sinapyl alcohol and 4-hydroxycinnamyl alcohol are the precursors for lignan synthesis.*P*-Coumaryl acid, ferulic acid and sinapic acid synthesized in the phenolic acid pathway lead to the formation of 4-hydroxycinnamyl alcohol, coniferyl alcohol and sinapyl alcohol, respectively. Formation of *p*-Coumaryl acid 4-hydroxycinnamyl alcohol, ferulic acid coniferyl alcohol, sinapic acid sinapyl alcohol are catalyzed by 4-hydroxycinnamate CoA and cinnamoyl CoA reductase. Coniferyl alcohol gives rise to the lignans with 9(9′) oxygens such as furofuran (pinoresinol, lariciresinol, secoisolariciresinol), dibenzylbutane, dibenzylbutyrolactone, aryltetralin, arylnaphthalene. On the other side, *p*-coumaric acid leads to the formation of the lignans without 9(9′) oxygen such as furan, dibenzocyclooctadiene, and dibenzylbutane [86] (Figure 2). Lignans are widely distributed in Gramineae, including cereals like wheat bran, rye bran, oats (*Avena sativa* L. Family: Poaceae), barley (*Hordeum vulgare* L. Family: Poaceae), triticale, and corn to name a few) [87]. Pumpkin (*Cucurbita pepo* L. Family: Cucurbitaceae), flax (*Linum usitatissimum* L. Family: Linaceae; richest source), sunflower (*Helianthus annus* L. Family: Asteraceae), poppy (*Papaver somniferum* L. Family: Papaveraceae), sesame (*Sesamum indicum* L. Family; Pedaliaceae) and oilseed crops are also observed to have high lignin content- mostly concentrated in the seeds [88].

## 4. Plant Defense Induction Mediated by Polyphenols

**Figure 3:**
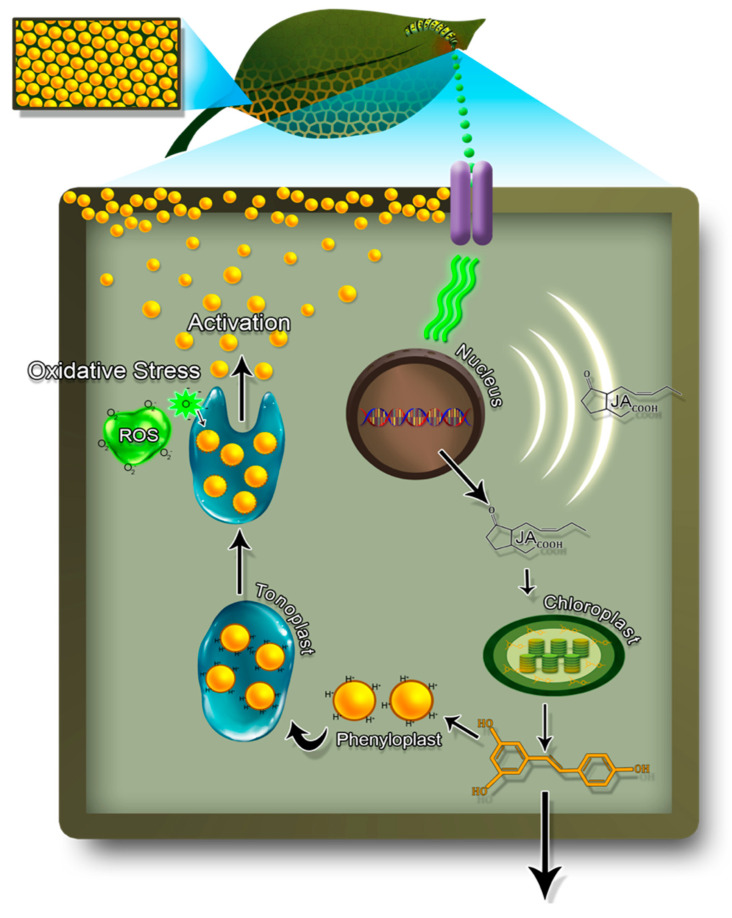
Schematic representation of signaling cascade after insect herbivory at the cellular level inside a plant cell. The regurgitant of caterpillar that includes contents from salivary gland and gut consists of fatty acid conjugates, β-glucose oxidase, peroxidase that acts as elicitors. Elicitors binds with the receptors on cell membrane and cause biochemical changes in the cell culminating in gene expression and the activation of octa-decanoid pathway [117] which upregulates defense- related genes followed by down-regulation of photosynthesis genes. The upregulation of defense genes that encodes proteins can be broadly classified into three categories- defense genes which produce anti-nutritional proteins and the enzymes involved in shikimate-phenylpropanoid pathway producing secondary metabolites, proteinase inhibitors which are involved in cross-linking and polymerization of cell walls and the third includes phytohormone signaling pathway genes for i.e., jasmonic acid, salicylic acid and ethylene. Jasmonic acid moves to plastid/chloroplast to activate the chief enzyme of shikimate pathway i.e., phenylalanine ammonia lyase (PAL). Most of the polyphenol biosynthesis takes place in plastids, however flavonoid production occurs either in the cytoplasm or the cytoplasmic surface of endoplasmic reticulum [127]. PAL in the stomata diverts amino acids from primary metabolism toward the formation of secondary metabolites including a diverse set of polyphenols via activation of a suite of defense genes [128]. Some polyphenols are shuttled outside the cell to act as anti-feedant or anti-deterrent to ward-off the herbivory, while others are stored inside the tonoplast of cells for quick-future action. These polyphenols are compartmentalized by converting them into inert and reduced state called phenyloplast, protected inside the tonoplast. During the successive herbivore attack, the regurgitant of herbivores that activates reactive oxygen species generates oxidative stress in the cell, leading to dissolution of compartments and release of polyphenol oxidase. Polyphenol oxidase can form quinones which act as anti-nutritional proteins interfering with digestibility and nutrient uptake of insects or produce proteinase-inhibitors leading to cross-linking and polymerization of cells leading to herbivore defense. Illustration by Annette Diaz, conceptualized by Ishveen Kaur and Japneet Kaur.

Herbivores attack plants by scratching, mining, chewing, biting, sucking, galling, wounding, parasitizing, and even evading the leaf surface using their secretions [4,100,101]. This attack in turn initiates a suite of defenses in form of physical (spines, trichomes and sclerophylly) and chemical defenses in plants [102,103,104] through signaling molecules [105], phytohormone pathways [106], and the initiation and synthesis of physical defense structures and/or formation of defensive chemical compounds such as alkaloids, [4] or activating the compounds already present in inactive form such as cyanogenic glycosides [107,108] and benzoxazinoids [109,110] to name a few (Figure 3). For example., two major weeds in the Solanaceae—*Solanum carolinense* L. and *Solanum elaeagnifolium* Cav.—possess a diverse suite of constitutive defenses including non-glandular stellate trichomes that negatively affect herbivore feeding [6], internode spines that deter herbivores [7]. More interestingly, these defense traits are also highly inducible post herbivory leading to a well-coordinated induced defense phenotype [111]. Induced defenses are also thought to be induced by elicitors produced by herbivores in their oral secretions. For example, the regurgitant of caterpillars or salivary secretions contain polyphenol oxidase, peroxidase and reductase which activate plant responses and signaling pathways ultimately leading to the production of polyphenolic compounds which are either toxic or repellent to herbivory [112,113] (Figure 3). These include oral secretions isolated from lepidopteran species (e.g., glucose oxidase from saliva of *H. zea* and β-glucosidase from *P. brassicae*), oviposition fluid contains elicitors in the form of long chain diols known as bruchins and the fatty-acid-amino-acid conjugates (FACs) found in the regurgitant of larvae of Sphingidae (Hawk moths), Noctuidae (cut worms) and Geometridae (inch worms) [114].

After recognizing these compounds, possibly through surface receptors [115], plants activate phytohormones such as jasmonic acid (JA), salicylic acid (SA) and ethylene which act as signaling molecules that spread throughout the plant apart from the wound site (local) to other plant parts (systemic) to induce various transcriptional factors and consequently, differential defense gene expression [112,114] (Figure 3). In their classic work, Farmer and Ryan showed that wound signaling speeds up JA production through octadecanoid pathway, which in turn activates the plant defense genes [116]. These compounds in general, are part of the octadecanoid defense pathway, primarily mediated by JA and methyl jasmonate [117,118] to produce herbivory induced secondary metabolites [119,120]. In tomato, expression level of polyphenol oxidase (PPO), proteinase inhibitor (PIs), and lipoxygenase (*LOX*) gene expression levels have been found to increase at local and systemic levels, regulated via octadecanoid pathway in response to wounding [121]. More specifically, the phytohormones produced through the octadecanoid pathway increases the expression of phenylalanine ammonia lyase (PAL; the chief enzyme necessary for regulation and operation of phenylpropanoid shikimate pathway), which in turn diverts amino acids from primary metabolism towards secondary metabolite production [122]. However, induction of signaling pathways is herbivore specific (e.g., feeding guild) and usually crosstalk is observed between different signaling molecules in the presence of multiple herbivory attack. This crosstalk can be either antagonistic or synergistic to utilize minimum resources to sustain plant growth and development, and to mount most effective defense strategy depending on the type of herbivores in action [123].

Most of these interactions between the hormones have been well understood at the molecular level [2,124]. For example, SA pathway induced by sucking pest and biotrophs through the regulatory protein NPR1 that also reduces the activity of JA. For instance, in tobacco (*Nicotiana tabacum* L.) SA induces defense response against *Tobacco mosaic virus* and JA against chewing herbivores [125] when plants are under multiple herbivores. JA is primarily induced by lipoxygenase (*LOX*) genes when plants are attacked by chewing herbivores [126]. Furthermore, it has also been observed that plants respond much faster to herbivory attack in comparison to mechanical damage, owing to the fact that some of the polyphenols produced after prior herbivory are stored in tonoplasts, and fast tracked for a rapid defense response (Figure 3).

## 5. Mode of Action of Polyphenol Mediated Defenses

**Figure 4:**
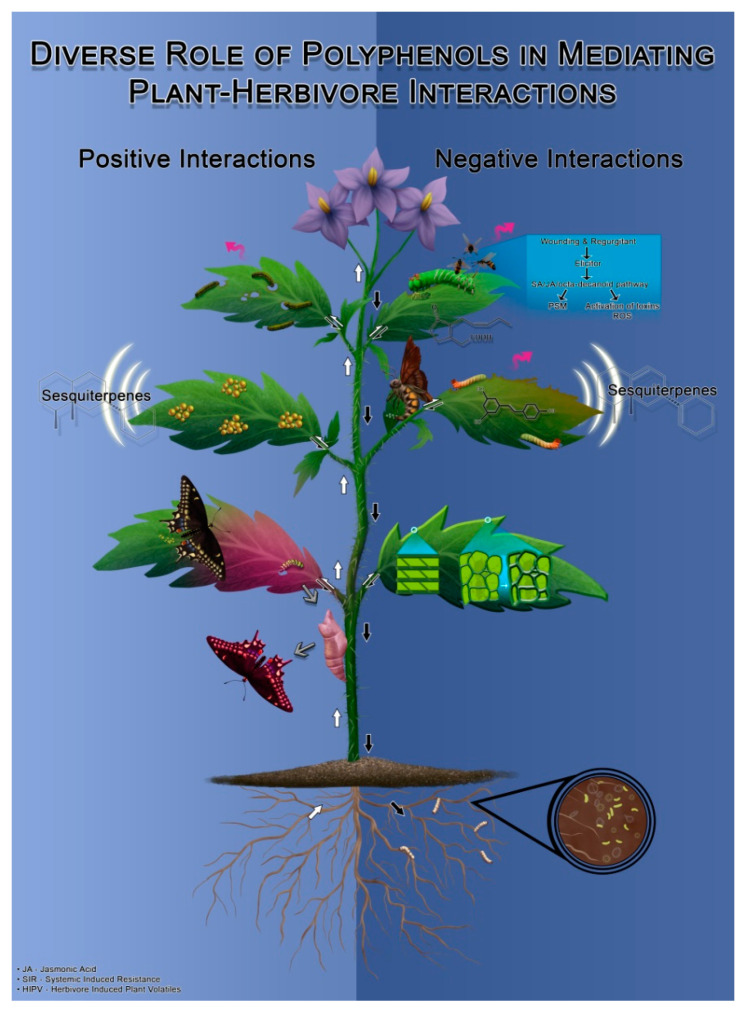
Schematic illustrating the wide gamut of herbivory related functions performed by polyphenols in plants. Plants produce polyphenols at the advent of adverse conditions such as biotic and abiotic stresses. Herbivory causes abrasions, wounds, and tissue loss which act as signal for the production of polyphenols. Moreover, the saliva or regurgitant from herbivores contain peroxidases which act as elicitors for the activation of different signaling pathways [112]. These secretions activate different plant hormones and signaling pathways such as jasmonic acid, salicylic acid and octadecanoid pathway [128] which generate and transmit signals to all the parts of plants which is depicted by bi-directional arrows running throughout the plant leading to generation of Systemic Induced Resistance (SIR) in the plants [148]. The production of polyphenols also leads to synthesis of sesquiterpenes for defense priming of the neighboring plants. Both positive and negative impacts are being illustrated on the plant with left and right sides of the schematic respectively. Polyphenols generally have anti-feedent and anti-deterrent effect on most of the insects. Flavonoids (class of polyphenols) and tannins have cascading effects on the feeding and oviposition activity of tobacco hornworm caterpillar [*Manduca sexta* L.; [8,22], also protects plants against the damaging herbivores by releasing herbivore-induced plant volatiles (HIPV) attracting their predators and parasitoids [149]. Activation of defense mechanisms also leads to the production of reactive oxygen species (ROS) which ultimately lead to the formation of polyphenol oxidase and subsequent synthesis of compounds such as proteinase inhibitors preventing the digestibility of tissues by cross-linking and polymerizing the cells walls with alkylated amino acids. Polymerization of cinnamyl alcohol into lignin by polyphenol oxidase (formed due to activation and synthesis of polyphenols) deposits lignin in leaves and fruits which also confers resistance to the plants [150]. These chemical toxins are also observed to have negative impacts on activity and functionality of microbes, thus indirectly affecting their symbiotic insects as well [151]. Polyphenols are also observed to affect the herbivores positively; thus, playing dual role in plant-insect relationship dynamics. Flavanone glycosides present in carrot (*Daucus carota* L.; Apiaceae) acts as oviposition stimulant for black swallowtail butterfly (*Papilio polyxenes* Fabricius) by releasing volatiles which attract the insects to lay eggs [145], and sequestration of flavone glucosides in the wings of lycaenid butterfly (*Polyommatus bellargus* Rottemburg) aiding them in visual communication and mate recognition. Illustration by Annette Diaz, conceptualized by Ishveen Kaur and Japneet Kaur.

Polyphenols affect herbivore growth by antibiosis, antixenosis or antisymbiosis modes of action. Antibiosis, in this context refers to the production of antibiotic compounds by the host plant inhibiting the growth, survival, development and reproduction, of insect herbivores [129] (Figure 3). As discussed earlier, insect herbivore regurgitants produced during feeding can alter defense gene expression, leading to the production of key defense compounds, including defense proteins and phytohormones. As a general mechanism, once an herbivore initiates feeding on plant tissue, amino acid polypeptides such as systemin can enhance the production of lipase enzyme in the receptor cell membrane leading to release of linolenic acid. Linolenic acid then acts as precursor of jasmonic acid signaling pathway, which ultimately produce peroxidase (PO), polyphenol oxidase (PPO) and proteinase inhibitors. They oxidize phenols to form reactive oxygen species and quinones [130,131,132]. Quinones act as anti-nutritional proteins interfering with digestibility and nutrient uptake of insects [133] (Figure 3 and Figure 4), causing significant reduction in herbivore fitness. These proteinase inhibitors also cause starvation in insects as they bind with the digestive enzymes inside insect midgut, reducing digestibility and release of nutrients and minerals required to perform essential metabolic functions for survival [12].

Polyphenols can also be toxic to insect herbivores by causing oxidative damage in the midgut through the following mechanisms. They readily bind with thiols, thereby reducing non-protein thiols and ascorbic acid both in plants as well as in the midgut epithelial tissue of midgut of herbivore when they consume polyphenols while feeding on plant, as examined during foliar feeding on soybean by *H. zea* [134]. The effect of binding of the polyphenols with the digestive enzymes of insects is reflected by the delay in the development, molting and consequently, reduction in fitness of insect herbivore. Rehman et al. (2013) reported the delay in development of mites due to the binding of phenolics to the digestive enzymes when fed on plant cultivars with high catechol content [135]. Flavonoids (apigenin, chrysin, luteolin and quercetin) have been also found to inhibit the EcR (ecdysone receptor) dependent gene expression in insects which affect their molting [136]. Stilbenes can inhibit the crocin and diphenyl picrylhydrazyl (DPPH)- insect growth regulatory enzymes’ activity in *S. frugiperda* J. E. Smith [51]. Flavonoids and phenolic acids (ferulic acid, vanillic acid and 4-hydroxybenzoic acid) have been also found to inhibit acetylcholinesterase (an enzyme involved in molting) in rice weevil (*Sitophilus oryzae* L.) [137]. Tannins are also reported to bind with the proteins and digestive enzymes in the gut of insect larvae causing similar effects. Similarly, binding of quinione to the dietary proteins has been found to be cause anti-nutritive in *M. sexta* [138], and quinones also undergo addition reaction with thiols and amino groups in digestive system of herbivores, which drastically reduces the availability of dietary protein [139]. Flavonoids such as taxifolin have been found to inhibit the activity of glutathione S-transferases enzyme (which detoxify insecticides) thereby, enhancing insecticidal properties [140,141]. Flavonoids have also been found to affect gustatory sensilla and their neuronal responses affecting food choice and consequently reducing consumption [142,143,144]. For example, rutin and quercetin 3-glucosylgalactoside present in soybean (*Glycine max* L.) leaves reduce the food consumption of cabbage looper (*Trichoplusia ni* Hubner) after detection [142,143]. And finally, in addition to inhibitory effects on digestion, stilbenes have been also reported to have anti-molting activity by acting as ecdysteroid receptor antagonist leading to premature induction of molting or even failure in molting [145,146,147].

Antixenosis or non-preference is the reduced preference for a host plant by herbivores, primarily due to the defense responses in plants that can affect their growth and development. For example, proteinase inhibitors produced after herbivory makes plants unpalatable and hence are not preferred by herbivores. Green and Ryan were the first to report the induction of proteinase inhibitors upon herbivory in promoting resistance in plants [152]. Dreyer and Jones demonstrated that flavonoids such as dihydrochalcones and polar phenolic compounds in wheat has strong anti-deterrent activity for green peach aphid (*Myzus persicae* Sulzer) and wheat aphid (*Shizaphis graminum* Rondani) [153]. Resistance to herbivory by antixenosis is also achieved by the morphological adaptations such as, hairiness, wax on leaves, color and by emitting foul-smelling volatile organic compounds such as terpenes [23,154,155]. Collectively, this reduces host location, oviposition activity, colonization, or have adverse post ingestive effects on insects [156]. Additionally, peroxidase (PO) enzyme regulates the defense signaling in plants leading to hypersensitive response, which also increases the lignification of cell wall, thereby reducing the digestibility of plant tissue. For example, peroxidase enzyme can modify the structure of polyphenols; peroxidase enzyme converts chlorogenic acid into chloroquinone, which binds to amino acids significantly reducing their availability [157], similar to quinones that attach to proteins and reduce their availability. Taken together, insect feeding on plants enhance the production of PO and PPO activity, which ultimately oxidize polyphenols and they act as either physical or chemical barriers or as signaling molecules providing resistance to plants against insect pests [132].

Antisymbiosis, on the other hand, is an example of the plant defense mechanism that indirectly affects growth of insect herbivores by affecting the growth and development of beneficial microbes associated with insects. Polyphenols have been shown to have antimicrobial activity on microbial symbionts of insect herbivores, thereby indirectly impacting herbivores. For example, condensed tannins were reported to repel leaf cutter ants (*Atta* spp.) by affecting the activity of wood rotting basidiomycete fungi in symbiosis with leaf cutter ants [151]. Tannins from Eurasian watermilfoil (*Mtriophyllum spicatum* L. Family: Haloragaceae) have been found to have allelochemical effect on the gut symbiont of water veneer (*Acentria ephemerella* Denis and Schiffermüller), thereby affecting their larval growth [158]. Although these examples provide an insight into the highly diverse and tightly regulated species-specific effects of polyphenols, investigating how different polyphenolic molecules act inside the insect body, their targets, and consequently their cellular and ecological effects is still a black box and needs further investigation.

## 6. Buffer-Storage of Polyphenols for Future Responses

Plants have also been found to sequester phenolic compounds in the cell vacuole, to swiftly combat any future attack [159,160,161]. These compounds are not only toxic to herbivores but are also found to be toxic to plants as well. Consequently, plants tend to store them in special compartments known as phenyloplasts; the cells of thylakoid membrane which are produced via redifferentiation of primary cells. In order to store polyphenols in these cells, they are first detoxified by conjugating it with glycosides to form phenylglycoside, making them hydrophilic to remove their toxicity [162]. These phenyloplasts are progressively filled with phenylglycosides until they become mature enough to be released outside the chloroplast [162]. Once filled with polyphenolic compounds, these molecules move inside the vacuoles. At the onset of herbivory, signaling molecules such as reactive oxygen species (ROS) are produced by insect oral secretions [105] leading to oxidative stress inside a cell, forcing phenyloplasts to break their outer protective layer, thus releasing various polyphenolic compounds. [162]. These compounds released from the vacuole, cross-link and/or polymerize cell wall, which imparts mechanical strength and rigidity to the plants, posing a harder barrier for herbivores to continue feeding by chewing and biting. PPO also oxides the secondary metabolites to form polymerized quinones [114] that act as proteinase inhibitors which bind with several essential enzymes inside the insect body, obstructing several essential physiological processes, digestive ability and nutritional uptake [114].

## 7. Defence Fitness Trade-Offs in Response to Insect Herbivory

Under continuous herbivory, plants reprogram their cellular machinery to allocate their energy resources among defense, growth and reproduction, in line with the predictions of resource allocation theory [163]. The theory predicts when plants possess limited resources to carry diverse functions, they reallocate their resources in a way that optimizes their overall performance, efficiency, vigor, and fitness [14,163,164]. As expected, polyphenolic secondary metabolites also mediate such trade-offs. For example, herbivory by *T. ni* on wild parsnip (*Pastinaca sativa* L; Apiaceae) plants, makes them divert their resources towards the production of furanocoumarin (polyphenols), thus causing scarcity of resources required for growth and development, observed as a drastic reduction in seed mass, and consequently fitness [165]. In another example., *Psychotria horizontalis* (Rubiaceae) plants exposed to pyralid and ctenuchid caterpillars induce the production of tannins leading to herbivore defenses at the cost of reduced growth of plants [166]. Post-herbivory, plants tend to overcome losses incurred during herbivory by enhancing metabolism and photosynthesis at the damaged site to invest in regrowth with possible fitness effects [167]. However, this leads to the diversion of resources towards defenses effectively reducing available resources-leading to shorter lifecycle and differences in root/shoot ratio. Even with all the recent developments in molecular genetics and metabolomics, we are still in our infancy on understanding the mechanisms underlying transgenerational effects (e.g., epigenetics) of herbivory, and how the intensity of herbivory influence the production and allocation of resources in their offspring. Being primarily mediated through polyphenols, studies on such trade-offs in resource reallocation between growth and defense examining single and groups of phenolic compounds can be used to explore more in this area, with an eye for potential application for crop improvement programs and pest management.

## 8. Recent Developments in Secondary Metabolite Research

It is clear that recent emphasis in this field is to elucidate the underlying molecular mechanisms involved in polyphenol biosynthesis. More recently, microRNA’s (non-coding, 21–24 nucleotides long RNA strands regulating gene expression) have been reported to be involved in the biosynthesis of PSM [168]. For instance, 4-coumarate-CoA ligase gene involved in flavonoid biosynthesis have been targeted by miR172i and caffeoyl-CoA O-methyl transferase involved in lignin biosynthesis targeted by miR1438 [169]. Also, by modifying the expression of such genes involved in biosynthetic pathway, methods to enhance their synthesis can be identified. Although exploration at a molecular level to alter the pathways to regulate the synthesis of polyphenolic compounds is still a work in progress, most of such research has been carried out to extract specific PSM for pharmaceutical and food industry with less focus on plant-insect interactions. More advancement is required in this area which will potentially assist in breeding plants that are naturally and sustainably resistant to various biotic and abiotic stresses.

Re-focusing to extract such compounds by developing inexpensive, simple, environmental and farmer friendly methods to combat the losses incurred due to biotic stressors. Recently, polyphenol rich pericarp extract, a byproduct of corn processing industry, extracted by inexpensive techniques has been found to negatively affect the growth and development of specialist insect herbivore *M. sexta* [8,23] (Table 1) as well as against generalist herbivore fall armyworm (*S. frugiperda*) [9] (Table 1). Such waste byproducts of food industry and other waste plant sources should be further explored under different systems and herbivores with different feeding behavior. Such developments do have the potential to become an alternative to the over reliance on synthetic pesticides in pest management.

## 9. Future Directions

Plant insect interactions and chemical ecology have benefitted greatly from understanding and quantifying secondary metabolites and examining their role in insect traits. Recent advances in molecular biology and metabolomics has taken this to the next level by allowing scientists to tease apart individual metabolites, genes and enzymes to target these specifically. Large number of enzymes and genes have been identified, which are mostly linked to different polyphenol pathways induced during plant defense responses to herbivory. While polyphenols have gained a lot of attention in parts- their use in food chemistry, they have also been an area of interest for studies in insect plant interactions. However, in this review we show how interspecific variation has an immense effect on the distribution and mode of action of different polyphenol compounds. Also, it is quite clear that a defensive compound in one plant system can be beneficial in another, reinforcing their species specificity. Theories of resource allocation and trade-offs in plants are still debated and requires better understanding to answer questions; when plants invest either in defense or in growth, when they start back investing in growth after threat from herbivory is over, and how various class of metabolites play a role in these tradeoffs’ decisions. The lack of studies in developing the methodologies of economically extracting these compounds to test them as potential biopesticide and to replace synthetic pesticides for sustainable crop production also warrants further investigation. Research in these areas will provide plant ecologists to further explore into defense mechanisms of plants and give insights to plant breeders for crop breeding. We urge our fellow scientists to move beyond model organisms and explore wild and native plant species and their interacting insects to understand, quantify, and extract PSM’s and examine in detail their role in mediating these interactions both in vivo and in vitro.

## Figures and Tables

**Table 1:** Major polyphenolic compounds present in plants mediating insect-plant interactions. These compounds are widely distributed in different plant parts, defending plants from insect herbivores and in some cases, increasing the survival of insect herbivores through multiple modes of action.

a. Polyphenols Mediated Defense Interactions with Insect Herbivores				
Compound	Plant	Insect Herbivore	Mode of Action	Reference
Anthocyanin and tannins (flavonoids)	Purple corn (*Z. mays*)	Fall armyworm (*Spodoptera frugiperda*)	Feeding deterrent	[9]
Genistein and rutin (flavonoids)	Soybean (*Glycine max)*	Stink bug (*Piezodorus guildinni)*	Antibiosis	[38]
Anthocyanin and tannins (flavonoids)	Purple corn (*Z. mays*)	Tobacco hornworm *(M. sexta)*	Ovipositional and feeding deterrent	[8,22]
Chlorogenic acid (phenolic acids)	Chrysanthemum (*Dendranthema grandiflora*)	Thrips	Pro-oxidant effect	[34]
*p*-Coumaric acid (phenolic acids)	Yellow maize (*Zea mays)*	Pink stalk borer (*Sesamia nanogriodes)*	Antibiosis	[39]
Chlorogenic acid (phenolic acids)	Yellow maize (*Z. mays*)	European corn borer *(Ostrinia nubilalis)*	Anti-feedant	[40]
Chlorogenic acid (phenolic acids)	Honeysuckle (*Lonicera maackii)*	Beet armyworm *(Spodoptera exigua)*	Feeding deterrent	[41]
Phenolic acids	European filbert *(Corylus* L).	Hazel aphid (*Myzocallis coryl)i*	Anti-feedant	[42]
Isoflavonoids (flavonoids)	Lupinus (*Lupin* spp.)	Grass grub (*Costelytra zealandica)* and African black beetle (*Heteronychus arator)*	Feeding deterrent	[43]
Piceid, isorhapontin, astringin.	Sakhalin spruce (*Picea glehnii)*	Japanese termite (*Reticulitermes speratus)*	Feeding deterrent	[44]
Syringic, coumaric, vanillic acid (phenolic acids)	Castor bean (*Ricinus communis* L.)	Castor semi-looper (*Achaea janata* L.)	Anti-feedant	[45]
Secoisolariciresinol, secoisolariciresinol diglucoside and (lignans)	Linseed (*Linum usitatissimum)*	Green peach aphid (*Myzus persicae)*	Toxic causing mortality	[46]
3-Deoxyanthocyanidin (flavonoid)	Sorgum (*Sorghum bicolor)*	Corn leaf aphid (*Rhopalosiphum maidis)*	Toxic causing mortality	[23]
Pisatin (flavonoid)	Pea (*Pisum sativum)*	Peaaphid (*Acyrthosiphon pisum)*	Feeding-deterrent	[35]
Quercetin dehydrate and rutin hydrate (flavonoid)	Apple (*Malus domestica)*	Wooly apple aphid (*Eriosoma lanigerum)*	Aphicidal	[47]
Vitisin B (stilbene)	Grape vine *(Vitis vinifera)*	African cotton leafworm (*Spodoptera littoralis)*	Chronic toxicity, anti-feedant.	[48]
Vanillic acid, syringic acid, cinnamic acid, and *p*-coumaric acids (phenolic acid)	Rice (*Oryza sativa*)	Yellow stem borer (*Scirpophaga incertulas),* leaf roller *(Cnaphalocrosis medinalis),* and brown plant hopper *(Nilaparvata lugens)*	Toxin	[18]
Ferulic acid	Rice (*O. sativa*)	Resistance against brown planthopper (*Nilaparvata lugens)*		[36]
Burchellin, podophyllotoxin, pinoresinol, sesamin, licarin A, or nordihydroguaiaretic acid (lignans)	Sesame (*Sesamum indicum*)*, Aniba burchelli*, chinaberry (*Melia azedarach*), Chaparral (*Larrea divaricate*) and Mayapple (*Podophyllum peltatum)*	Triatomid bug (*Rhodnius prolixus)*	Anti-molting	[49]
Pinoresinol + podophyllotoxin derivatives (lignans)	Chinaberry (*M. azedarach)*	Milkweed bug (*Oncopeltus fasciatus)*	Anti-molting	[50]
Combretastatin A-4, 4,4′-dihydroxystilbene, resveratrol and 3,3′,5,5′-tetrahydroxy-4-methoxystilbene	Zote (*Yucca persicola)*	Fall armyworm (*S. frugiperda)*	Toxin	[51]
Caffeic acid and chlorogenic acid	Cotton (*Gossypium hirsutum*)	Corn earworm (*Helicoverpa zea)*	Arrest the larval growth and development	[52]
Vitisin A and vitisin B (stilbene)	Grapes *(Vitis vinifera)*	Colorado potato beetle *(Leptinotarsa decemlineata)*	Inhibit larval growth, chronic toxicity and anti-feedant	[53]
**b. Polyphenols Mediated Interactions with Insect Herbivores that Enhance Herbivore Traits**				
Phenolic glucosides and tannins	Almond willow *(Salix triandra* L.)	Shrank leaf beetle (*Gonioctena linnaeana)*	Feeding stimulant	[54]
Phenolic glucoside (tremulacin 1.5%)	Willow *(Salix rosmarinifolia)*	Shoot gallow sawfly *(Euura lasiolepis)*	Oviposition stimulant	[55]
Isoquercitrin, quercetin and quercetin-3-methyl ether	Chickpea *(Cajanus cajan)*	Cotton bollworm *(Helicoverpa armigera)*	Feeding stimulant	[56]
Flavonoids	Milkweed (*Ascelpias curassavica* L.)	Monarch butterfly *(Danaus plexippus*)	Oviposition stimulant	[57]
Flavonoid glycoside, rutin (pentahydroxyflavone-3-rutinoside	Lettuce (*Lactuca sativa)*	American grasshopper *(Schistocerca americana)*	Feeding stimulant	[58]
Quercitrin, iso- quercitrin and rutin (flavonoid)	Cotton (*G. hirsutum)*	Corn earworm *(Heliothis zea)*	Feeding stimulant	[59]
Flavanol glycosides and quercetin	Narrow leaf wedge (*Vicia angustfolia* L.)	Bean aphid (*Megoura crassicauda*)	Stimulate probing	[60]
Flavonoids (aglycones, quercetin and myricetin	Crown vetch *(Coronilla varia)* and Alfalfa *(Medicago sativa)*	Blue butterfly *(Polyommatus icarus)*	Sequestration in wings (mate recognition)	[61]
Flavonoids	Mulberry (*Morus alba)*	Silk moth *(Bombyx mori)*	Sequestration in pupae	[62]
Flavone C-glycosides	Crown vetch *(Coronilla varia)*	Larvae of lycaenid butterfly (*Polyommatus bellargus)*	Sequestration in wings	[63]
Flavone glycoside, luteolin glycoside	Carrot *(Daucas carota)*	Black swallowtail butterfly (*Papilio polyxenes)*	Oviposition stimulant	[64]
Quercetin and rutin	Milkweeds (*Asclepias curassavica*)	Female monarch butterfly (*Danaus plexippus)*	Oviposition stimulant	[65]
Flavonoid glycosides	St John’s Wort (*Hypericum* Spp.)	Saw fly (*Tenthredo zonula*)	Sequester compounds in larval body.	[66]
Flavonoids	Kale (*Brassica oleracea* var. *acephala*)	*Cabbage butterfly (Pieris brassica)*	Sequestration	[67]
*trans*-Chlorogenic acid	Wild parsnip, (*Pastinaca sativa*),	*Black swallowtail butterfly(P. polyxenes)*	Oviposition stimulant	[68]

**Table 2:** Main classes of polyphenols and their localization in different plant parts.

Plant	Plant Part	Types of Compounds	Reference
Rice (*Oryza sativa*)	Rice straw	Phenolic acids (*p*-hydroxybenzoic, vanillic, coumaric, syringic, ferulic acid)	[89]
Soybean (*Glycine max*)	Seed	Phenolic acids (syringic, ferulic and vanillic acids)	[90]
Cotton *(Gossypium hirsutum)* L.	Leaves	Phenolic acid (gallic acid, catechin and caffeic acid)	[91]
Sunflower (*Helianthus annus*)	Seed	Phenolic acid (chlorogenic acid)	[92]
Citrus fruits, apple, berries, peaches, fruits, nuts, berries, tea, red wine	Fruit	Flavonoids (flavanols)	[93,94]
Red rose (*Rosa indica),* China rose (*Hibiscus rosachinensis*),	Flowers	Flavonoids (anthocyanins)	[95]
Rice bran	Flowers	Flavonoids (flavone)	[96]
Soybean, alfalfa, red clover, chickpeas, peanut	Seeds and vegetables	Flavonoids (isoflavones)	[97]
Tea leaves (black tea and oolong tea)	Leaves	Flavonoids (catechins)	[98]
Sesame *(Sesamum indicum)*	Seed	Lignan (furofuran lignan)	[88]
Tea (*Thea* sp.)	Leaves	Lignans (matairesinol and secoisolariciresinol)	[99]
Conifers	Roots, bark and needles	Stilbene (*trans*-astringin and *trans*-isorhapontin)	[81]

## Abbreviations

MDPI	Multidisciplinary Digital Publishing Institute
DOAJ	Directory of open access journals
TLA	Three letter acronym
LD	Linear dichroism
